# Independent losses of a xenobiotic receptor across teleost evolution

**DOI:** 10.1038/s41598-018-28498-4

**Published:** 2018-07-10

**Authors:** Marta Eide, Halfdan Rydbeck, Ole K. Tørresen, Roger Lille-Langøy, Pål Puntervoll, Jared V. Goldstone, Kjetill S. Jakobsen, John Stegeman, Anders Goksøyr, Odd A. Karlsen

**Affiliations:** 1 Department of Biological Sciences, University of Bergen, Bergen, Norway; 2 Centre for Ecological and Evolutionary Synthesis (CEES), Department of Biosciences, University of Oslo, Oslo, Norway; 3 Centre for Applied Biotechnology, Uni Research Environment, Bergen, Norway; 4 Biology Department MS, Woods Hole Oceanographic Institution, Woods Hole, MA USA

## Abstract

Sensitivity to environmental stressors largely depend on the genetic complement of the organism. Recent sequencing and assembly of teleost fish genomes enable us to trace the evolution of defense genes in the largest and most diverse group of vertebrates. Through genomic searches and in-depth analysis of gene loci in 76 teleost genomes, we show here that the xenosensor pregnane X receptor (Pxr, Nr1i2) is absent in more than half of these species. Notably, out of the 27 genome assemblies that belong to the Gadiformes order, the *pxr* gene was only retained in the Merluccidae family (hakes) and Pelagic cod (*Melanonus zugmayeri*). As an important receptor for a wide range of drugs and environmental pollutants, vertebrate PXR regulate the transcription of a number of genes involved in the biotransformation of xenobiotics, including cytochrome P450 enzymes (CYP). In the absence of Pxr, we suggest that the aryl hydrocarbon receptor (Ahr) have evolved an extended regulatory role by governing the expression of certain Pxr target genes, such as *cyp3a*, in Atlantic cod (*Gadus morhua*). However, as several independent losses of *pxr* have occurred during teleost evolution, other lineages and species may have adapted alternative compensating mechanisms for controlling crucial cellular defense mechanisms.

## Introduction

Teleost fishes represent the largest and most diverse vertebrate clade. By inhabiting a wide range of oceanic and freshwater habitats, ranging from tropical to arctic regions, the genetic composition and phenotype of teleost fishes have evolved accordingly^1,2^. However, genes involved in the orchestrated cellular recognition, biotransformation and elimination of chemical compounds, denoted the chemical defensome, are largely conserved through evolution^3–5^. Today, the increasing burden of man-made compounds poses a great risk to the aquatic environment. Since large pelagic and benthic teleost fish are the dominant group of predators in global oceans, they are particularly vulnerable to ecosystem-wide changes in energy resources^6^, such as those caused by pollution. Thus, understanding the genomic defense mechanisms and their downstream effects in these species is paramount in conserving environmental diversity.

Atlantic cod (*Gadus morhua*) was the first commercially important, large teleost species of which the genome was sequenced and assembled^7^. Furthermore, it is commonly used as an indicator species in environmental monitoring programs and is an emerging marine model organism in toxicological studies^8–11^. Due to its habitats near offshore oil platforms, petroleum recovery facilities and coastal industries, the effects of contaminants in Atlantic cod are of great interest^12–15^. We have recently mapped the full suite of cod cytochrome p450 (*cyp*) genes, providing further clues to the inherent capabilities for biotransformation of endogenous and xenobiotic compounds in this species^16^. However, the proteins that regulate various chemical defense mechanisms have not been described in Atlantic cod.

Pregnane X receptor (PXR, NR1I2) and constitutive androstane receptor (CAR, NR1I3) are considered two of the main xenobiotic receptors in mammals that, in addition to binding endogenous steroid hormones, are activated by a structurally diverse range of drugs and environmental pollutants. Primarily expressed in the liver and small intestine, these xenosensors regulate the transcription of biotransformation enzymes, such as cytochrome P450 3A (CYP3A), and are thus central in orchestrating the chemical defensome. PXR and CAR belong to the large family of ligand-activated transcription factors known as nuclear receptors (NR), more specifically the subfamily NR1I, and show the common NR structural organization of a highly conserved DNA-binding domain (DBD) and a less conserved ligand-binding domain (LBD)^17^. The early evolutionary history of this subfamily is not clear, but the *NR1I* genes are hypothesized to diverge from a common ancestral gene, such as that identified in the chordate invertebrate *Ciona intestinalis*^18^. A vitamin D receptor (VDR, NR1I1) has been cloned from sea lamprey (*Petromyzon marinus*), an ancient vertebrate lacking calcified skeleton and jaws, and proposed to function in part as a xenosensor^19^. However, early in, or before, vertebrate evolution, this ancestral gene diverged into VDR and PXR^18^.

Phylogenetic analyses suggest that the further divergence of CAR and PXR occurred in a whole genome duplication (WGD) in, or prior to, the formation of the vertebrate crown lineage, Sarcopterygii^20^. However, except for mammals, which have retained both xenosensors, several independent losses of CAR and PXR have occurred in vertebrate evolution, leaving birds and lizards with CAR (also known as CXR^21^) and teleost species with PXR^22–24^. The aryl hydrocarbon receptor (Ahr) is another important transcription factor in the chemical defensome of vertebrates. Also known as the “dioxin receptor”, the ligands of this basic-Helix-Loop-Helix-PAS domain receptor include environmental pollutants such as 2,3,7,8-tetrachlorodibenzo-*p*-dioxin (TCDD) and certain planar polycyclic aromatic hydrocarbons like benzo[*a*]pyrene. The most studied piscine Ahr target gene is *cyp1a*^12,25^, but recent studies have indicated its co-regulative role in mediating transcription of the Pxr target gene, *cyp3a65*, in zebrafish^26–28^.

The aim of the present study was to examine the evolution of Pxr orthologs in teleost fish. Importantly, based on global genomic searches and in-depth analysis of gene loci of 76 teleost genome assemblies^29,30^, we show that several independent losses of *pxr* have occurred during piscine evolution. Furthermore, although we show that a *pxr* gene is absent in most species in the Gadiformes order, we detect its presence in the Merluccidae (hakes) family. Moreover, by mapping the complete NR complement in Atlantic cod, we show that the other nuclear receptors are conserved in this Gadiform species. Finally, lack of Pxr agonist-induced transcriptional response in Atlantic cod liver, as well as a highly increased number of putative Ahr response elements in the promoter region of Pxr target genes, suggest that Ahr has evolved a compensatory role in the absence of Pxr in this species. However, as several independent losses must have occurred during teleost evolution, other lineages may have adapted other compensating mechanisms. This is the first report with compelling evidence for multiple independent losses of the *pxr* gene in vertebrate evolution and accentuate the need for understanding how the chemical defensome system has evolved in teleost fish.

## Results

**Figure 1:**
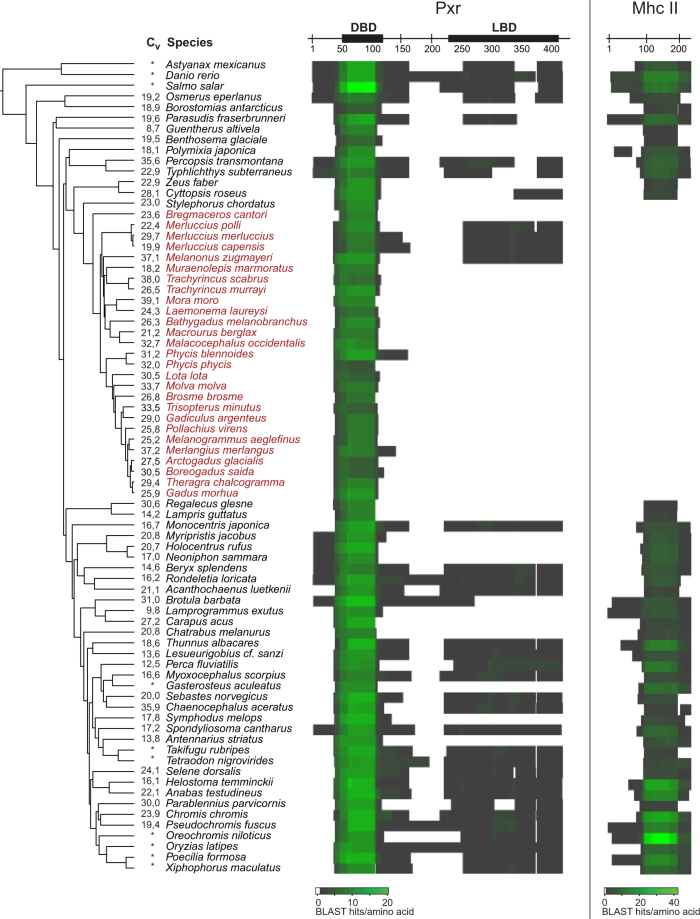
*In silico* searches using zebrafish pregnane x receptor (Pxr, 430 amino acids) show the loss of the *nr1i2* gene in many of the Gadiform species, as well as other teleost fish. Searches were iterated using zebrafish major histocompability class II (Mhc II, 234 amino acids) as a control. Each row represents the resulting coverage vectors of the *pxr* or *mhcii* BLAST hits identified in the query amino acid sequence in 76 different fish species. The DNA-binding domain (DBD) and ligand-binding domain (LBD) in zebrafish Pxr are indicated. The C_v_ values are average coverage of each nucleotide in the assemblies used from Malmstrøm, *et al*.^29^ and Malmstrøm *et al*.^70^, and asterisk indicate use of genomes available from ENSEMBL. The species are placed into in the same phylogeny as described in Malmstrøm, *et al*.^29^ and species belonging to the Gadiformes order are denoted in red.

*In silico* searches in 76 sequenced and assembled teleost genomes and using zebrafish Pxr as query sequence, showed that a *pxr* gene could not be identified in the majority of the fish species (Fig. 1). Additional searches using Pxr from medaka or tetraodon gave similar results (Supplementary Fig. 1). Surprisingly, *pxr* was only present in 33 out of the 76 genomes analyzed. Searches performed using zebrafish Vdrα as query, however, showed that a *vdr* gene was present in all species (Supplementary Fig. 2).

As a positive control of the pipeline used for the gene mapping, searches using zebrafish Mhc II were included, showing an identical phylogenetic distribution as published previously (Fig. 1)^29^. Multiple hits on the NR DBD are the result of the high similarity between these domains in the different NR proteins within a genome.

**Figure 2:**
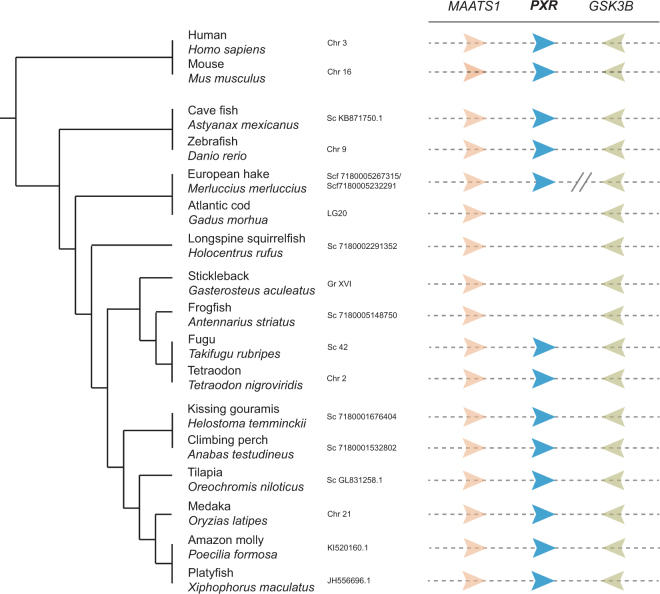
Schematic representation of the pregnane x receptor (*pxr*) syntenic regions in sequenced teleost genomes. The presence or absence of teleost *pxr* was confirmed in genomes where *MYCBP-associated*, *testis expressed 1* (*MAATS1*) and *glycogen synthase kinase 3 beta* (*GSK3B*) were identified and assembled to the same scaffold. The *Merluccius merluccius gsk3b* gene was located on a separate scaffold as *maats1* and *pxr*. Chr, chromosome; Sc, scaffold; LG, linkage group; Gr, group. The phylogeny is based on Malmstrøm, *et al*.^29^.

Furthermore, we analyzed the genomic scaffolds containing *MAATS1* and *GSK3B*, which flank *PXR* in all examined phyla that possess *PXR*. Figure 2 shows that the syntenic relationship of these genes is also maintained throughout the evolution of fishes. Importantly, in Atlantic cod and other species where *pxr* was not identified, no gene is present in the genomic region between *maats1* and *gsk3b*. Our results show that the loss of *pxr* has occurred in several orders, including Zeiformes, Holocentriformes, and Beryciformes. Importantly, out of the 27 genome assemblies in the Gadiform order, we only identified sequences similar to Pxr in the Merluccidae family (hakes, represented by *M*. *polli*, *M*. *merluccius*, and *M*. *capensis*) and *Melanonus zugmayeri* (Pelagic cod). The syntenic relationship of *maats1* and *pxr* was confirmed in European hake (*M*. *merluccius*), while *gsk3b* was assembled to another scaffold **(**Fig. 2**)**.

**Figure 3:**
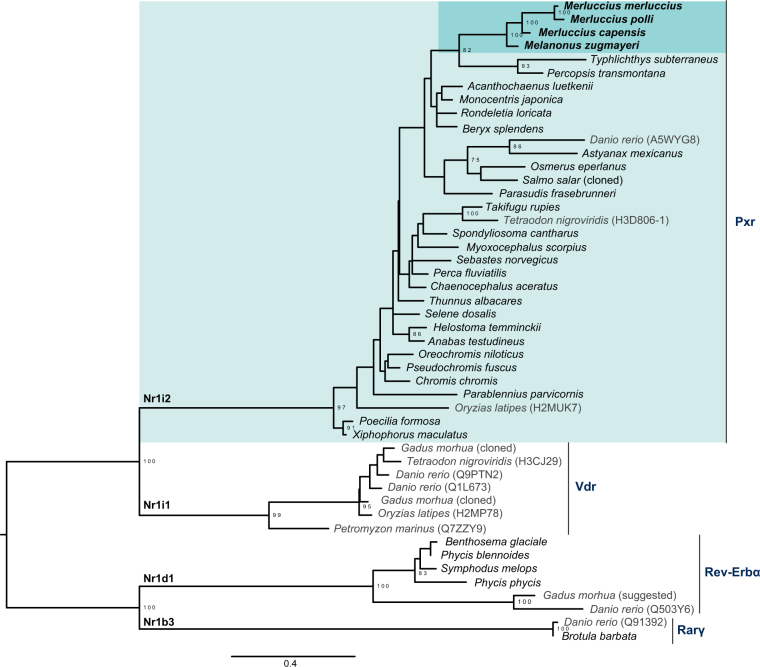
Phylogenetic relationship of teleost Nr1d and Nr1i nuclear receptor subfamilies. Based on their ligand binding domains, sequences identified in the bioinformatic searches using zebrafish, medaka, and tetraodon Pxr as query were aligned with annotated or cloned teleost Pxr (Nr1i1), vitamin D receptor (Nr1i2), retinoic acid receptor (Nr1b3) and Rev-ERbα (Nr1d1) (shown in grey), using Clustal Omega v1.2.2 with default parameters. The maximum likelihood phylogeny was analyzed in RAxML v 8.1.14 using the CATLG model, with 200 bootstrap replicates, and drawn in FigTree v.1.4.2. The figure was prepared in Adobe Illustrator CS5. Bootstrap values over 75 are shown.

In order to explore whether the presence of Merluccidae and Melanonidae *pxr* was due to retention or introgression, we aligned the translated LBDs of the identified Pxr sequences. Since Vdr (Nr1i1) are in the same NR subfamily as Pxr, we also included known teleost orthologs of this receptor, as well as orthologous retinoic acid receptor (Rarγ, Nr1b3) and Rev-Era (Nr1d1) sequences as an outgroup. Although the families are distantly related, the sequences identified from Melanonus and Merluccius genomes are grouped together in the resulting phylogenetic tree, showing that their Pxr sequences are specific for the Gadiformes order (Fig. 3). The phylogeny also supported our initial bioinformatic searches, as none of the Pxr sequences grouped with the teleost Vdr sequences. However, some short sequences that were identified using medaka and tetraodon Pxr as query (Supplementary Fig. 1) were found to group with Rarγ and Rev-Erbα (Nr1d1), and not Pxr (Fig. 3). While loss of the third member of the NR1I subgroup in mammals, CAR, is demonstrated previously in teleost fish^22,31^, we also performed searches using the unusual *Xenopus laevis* BXR^32^ without identifying any new Pxr-similar sequences.

**Figure 4:**
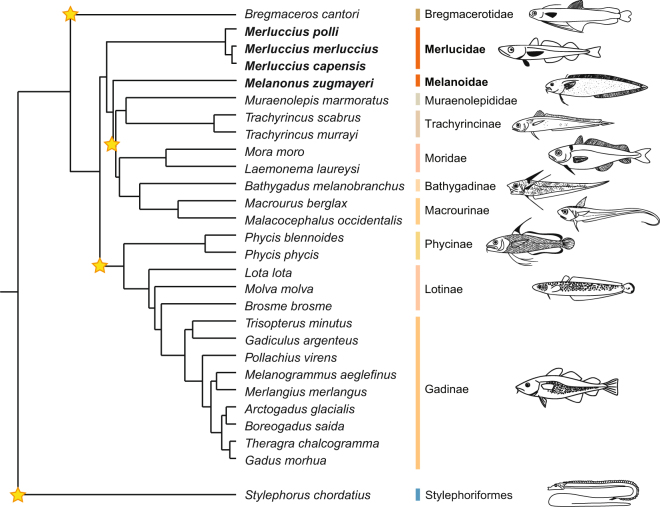
Early, independent losses of pregnane x receptor (*pxr*, *nr1i2*) in the Gadiformes order. The hypothesized evolutionary losses of *pxr* in Gadiform species are indicated by stars, leaving the gene retained only in the Merluccidae and Melanonidae families. Stylephoriformes is used as an outgroup, showing that a loss of *pxr* in this group precedes the multiple and independent losses in gadiformes lineage. The teleost illustrations by G. Holm and the phylogenic relationship are both from Malmstrøm, *et al*.^29^.

Thus, our investigations support the hypothesis that Pxr is retained in Merluccidae and Melanonidae even though several, possibly three, losses of *pxr* have occurred in the evolution of the Gadiformes order alone (Fig. 4).

**Figure 5:**
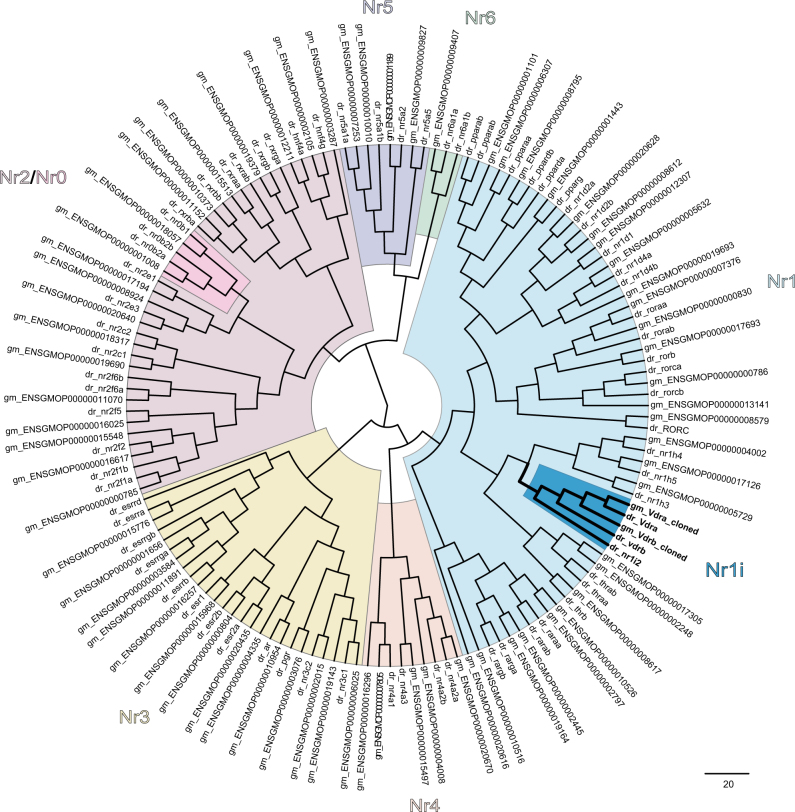
Pregnane x receptor (Pxr, Nr1i2) is absent in the Nr1i subgroup of nuclear receptors in Atlantic cod. In contrast, zebrafish has both Pxr (Nr1i2) and vitamin D_3_ receptor (Vdr, Nr1i1) paralogs in the highlighted Nr1i subgroup. The nuclear receptor protein sequences identified by HMM and Pfam profiles in Atlantic cod (*Gadus morhua*, gm_ENSEMBL protein id) and zebrafish (*Danio rerio*, dr_ENSEMBL gene name) were aligned using ClustalX. Cloned sequences of cod Vdra and Vdrb were also included. The phylogenetic tree was made with MrBayes and drawn in FigTree. Nr0–6 indicate the seven different subfamilies of nuclear receptors.

In the Atlantic cod genome (gadMor1^7^), HMM-searches identified 72 nuclear receptor-encoding genes, which were further mapped to 50 unique members of the NR superfamily (Supplementary Table 2). To visualize their phylogenetic relationship and distribution into the NR families and subfamilies, the NR complement of Atlantic cod and zebrafish were compared by multiple sequence alignments and visualized as a cladogram **(**Fig. 5**)**. Importantly, whereas the zebrafish NR1I subfamily contains Pxr (Nr1i2) and two paralogous Vdr proteins (Nr1i1a and Nr1i1b, denoted Vdrα and Vdrβ), only Vdrα and Vdrβ orthologs were identified in the cod genome. Furthermore, the cod genome holds additional orthologs to defensome-related NRs that are not identified in the zebrafish genome, including retinoic acid receptors (i.e. two *nr1b2* genes, and one *nr1b3*), hepatocyte nuclear factor 4a (*nr2a1*), glucocorticoid receptor (*nr3c1*), and androgen receptor (*nr3c4*).

Searches in the second version of the Atlantic cod genome assembly (gadMor2^33^) supported the absence of a *pxr* gene. Moreover, we made several attempts to amplify a *pxr* transcript from cod liver cDNA through polymerase chain reactions (PCR) using CODEHOP primers (Supplementary Table 3). These primers have previously been used to amplify *pxr* in other fish species, including Atlantic herring (*Clupea harengus*), Atlantic wolfish (*Anarhichas lupus*) and European eel (*Anguilla anguilla*) (Supplementary Table 5). In line with the results from the bioinformatic searches, we were also able to successfully amplify *pxr* from the Gadiform species European hake (*Merluccius merluccius*) (Supplementary Table 5). In contrast, none of the primers amplified sequences that matched *pxr* orthologs when using Atlantic cod cDNA as template.

**Figure 6:**
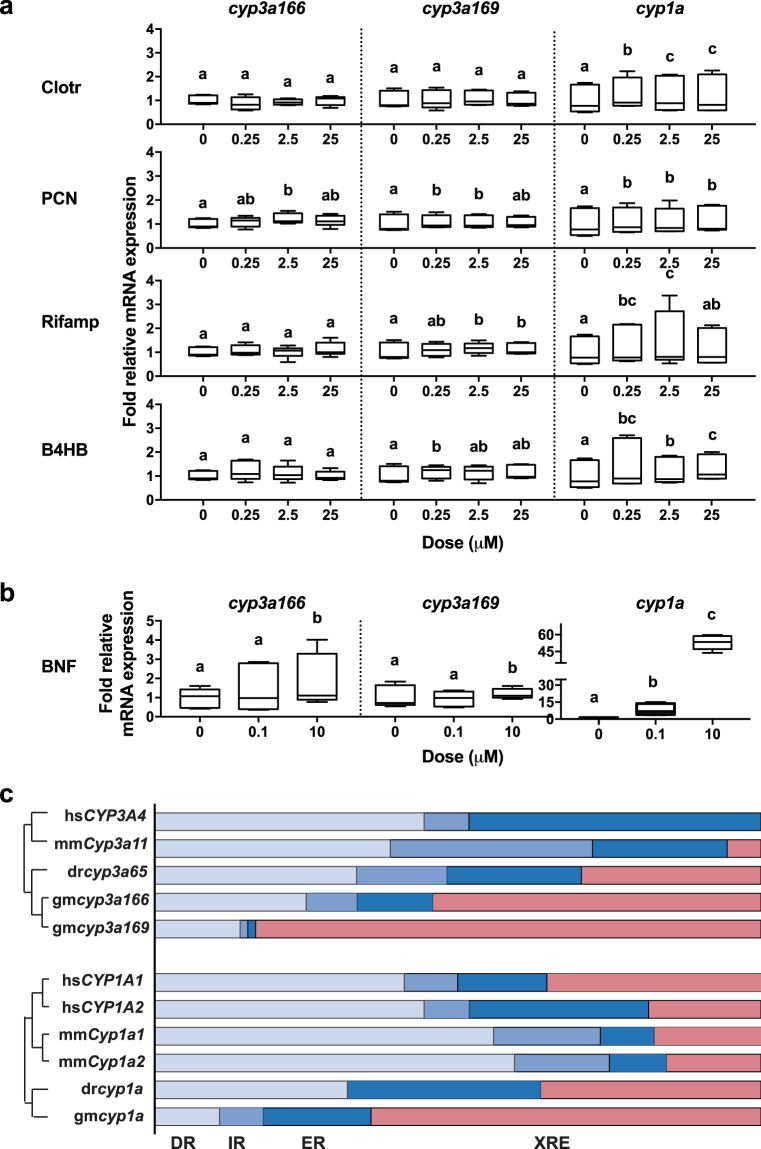
*Ex vivo* liver exposure studies and promoter analysis suggest that in Atlantic cod (*Gadus morhua*), the aryl hydrocarbon receptor (Ahr) is involved in the transcriptional regulation of well-known pregnane x receptor (*PXR*) target genes. Precision-cut cod liver slices was exposed for 24 h to well-known ligands of PXR (**a**): clotrimazole (Clotr), pregnenolone 16α-carbonitrile (PCN), rifampicin (Rif), butyl 4-hydroxybenzoate (B4HB), and Ahr (**b**): β-naphthoflavone (BNF), before relative transcription of *cyp3a166*, *cyp3a169*, and *cyp1a* was measured using real-time quantitative PCR. Acidic ribosomal protein and ubiquitin was included as reference genes. The results are from three individual experiments, each with three technical replicates (*n* = 9). The different letters (**a**–**c**) indicate significant differences (*p* < 0.05) in gene transcription, as calculated on log2-transformed data in a Standard least square model combined with a Tukeys *post hoc* analysis. Relative composition of response elements (**c**) in 13 kb upstream regions of *CYP3A* and *CYP1A* orthologs. Species included are humans (hs), mouse (mm), zebrafish (*Danio rerio*, dr), and Atlantic cod (*Gadus morhua*, gm). Potential nuclear receptor response elements were revealed using NHR-Scan, and the AHR-binding xenobiotic response elements (XREs) were identified by searching for the consensus sequence ‘KNGCGTGM’.

To study the transcriptional effect on the biotransformation genes *cyp3a* and *cyp1a*, we exposed precision-cut liver slices (PCLS) from Atlantic cod to a selected set of compounds known to activate mammalian and piscine PXR, and cod Ahr (BNF). The results show that the model zebrafish Pxr agonist, clotrimazole, had no significant effect on the transcription of *cyp3a169* and *cyp3a166*, but it did induce transcription of *cyp1a* (*p* < 0.05) (Fig. 6a). Transcription of all genes were slightly, but significantly (*p* < 0.05) affected by the mouse PXR agonist, PCN, whereas the human PXR agonist, rifampicin, and the medaka Pxr agonist, B4HB, significantly (*p* < 0.05) affected transcription of both *cyp3a169* and *cyp1a* (Fig. 6a). Although slightly more induced than the *cyp3a* genes, the induction of *cyp1a* was much less following exposure to these compounds, compared to the exposure with the Ahr agonist, BNF (Fig. 6b). The highest dose of BNF also affected the transcription of both *cyp3a* genes, but the mean fold change remained low. Finally, we mapped the XRE and putative NR response elements (REs) in a 13,000 base-pair region upstream of the transcription start sites of human, mouse, zebrafish, and Atlantic cod *CYP3A* and *CYP1A* orthologs. These results show that the three active PXR-specific REs determined for human *CYP3A4*^34^ are not conserved between these species, including mouse (Supplementary Fig. 2). The clusters of known XREs in human *CYP1A1*, mouse *Cyp1a1* and zebrafish *cyp1a* orthologs^35–37^, however, appear to be conserved and coincide position-wise. Importantly, whereas NR REs dominate the promoter regions of mammalian *CYP3A* genes, the promoter regions of the fish *cyp3a* orthologs have a higher number of potential XREs (Fig. 6c). Furthermore, the promoter regions of cod *cyp3a166*, *cyp3a169*, and *cyp1a* contain almost twice the number of putative XREs than zebrafish (Fig. 6c).

## Discussion

In the present work, we have demonstrated that multiple losses of *pxr* have occurred during teleost evolution. Through global genomic searches and in-depth analysis of gene loci of 76 teleost genome assemblies, we show that only 33 of these species have retained a *pxr* gene. Notably, our results show that within the Gadiformes order, only the Merluccidae family (hake) and *Melanonus zugmaeri* (Pelagic cod) have retained *pxr*, whereas the 23 other gadiform species represented have lost the gene. Based on current phylogeny of Malmstrøm, *et al*.^29^, Merluccidae and Melanonidae are on separate branches deep within the Gadiformes order, and have arisen later in evolution than *Bregmaceros cantori* (striped codlet) but earlier than Macrouridae (grenadiers) and *Muraenolepis marmorata* (marbled moray cod), which all have lost the *pxr* gene. Thus, the presence of *pxr* in Merlucciudae and Melanonidae could be due to retention of an ancestral gene together with several independent losses in the other Gadiform species, or it could have been reintroduced through introgression. As we demonstrate through the conserved syntenic relationship of the genomic region, and the phylogenetic pattern of the LBD of Merluccidae and *Melanonus zugmayeri* Pxrs, it is most likely that an ancestral gene is retained in these species. Moreover, based on current phylogeny, this means that multiple, independent losses have occurred in evolution of the Gadiformes order alone.

In other vertebrate species, PXR is an important xenobiotic receptor that governs much of the transcriptional response to chemical stressors. For instance, human PXR directly regulate expression of CYP3A isozymes, which are involved in the metabolism of more than 50% of all prescription medicines^38^. *Cyp3a* genes have been cloned and functionally characterized in several fish species^39–45^, but, with the exception of zebrafish^28^, the transcriptional regulation of teleost *cyp3a* genes remains largely unexplored. However, exposure to prototypical Pxr agonists resulted in upregulated transcription of *cyp3a* genes in Atlantic killifish (*Fundulus heteroclitus*), zebrafish liver and rainbow trout (*Oncorhynchus mykiss*)^46–48^. Furthermore, expression patterns of the subfamilies *cyp3b*, *cyp3c*, and *cyp3d* in ray-finned fishes (Actinopterygii) suggest involvement in xenobiotic metabolism, but this role has not yet been demonstrated^11,49–51^. Thus, the widespread absence of *pxr* orthologs in teleosts, and particularly among Gadiform species, raise interesting questions on how transcriptional regulation of biotransformation enzymes in these species has evolved.

By using Atlantic cod (*Gadus morhua*) as a representative of a teleost without *pxr*, we mapped the full complement of NR genes and show that Pxr is absent in the Nr1i subgroup. In line with the genome mining and syntheny analysis, we were unable to amplify a *pxr* gene from cod liver using CODEHOP primers. Notably, we were successful in using the primers to amplify a *pxr* ortholog from European hake (*Merluccius merluccius*), one of the few Gadiform species that have retained this gene.

Furthermore, we explored how transcription of cod *cyp3a* and *cyp1a* genes were affected by exposure to classical Pxr agonists. Exposure of cod PCLS to potent *in vitro* activators of human, mouse, zebrafish, and medaka PXR/Pxr, including rifampicin, PCN, clotrimazole, and p-hydroxybenzoic acid, respectively^52^, had little or no effect on *cyp3a* transcript levels. Furthermore, although exposure to the established cod Ahr agonist BNF had a significant effect on *cyp3a* transcription at the highest dose, the fold increase was very low compared to the BNF induction of *cyp1a*. Similarly, in an early study of Atlantic cod, hepatic distribution of a CYP3A-like isozyme P450b remained unaffected after *in vivo* exposure to BNF^53^. In mice, basal levels of *Cyp3a11* expression was elevated approximately 4-fold in the livers of PXR knockouts relative to their wild-type counterparts^54^, suggesting that PXR also has a repressing transcriptional role. We recently found a significant decrease in cod *cyp3a* expression in PCLS following a 24-hour exposure to 10 μM ethynylestradiol^55^. This is in line with previous studies that indicate that xenoestrogens affect the expression and activity of Cyp3a and Cyp1a in Atlantic cod and Atlantic salmon (*Salmo salar*)^8,56,57^, and suggests that the estrogen receptor (Er) signaling pathway also is involved in controlling *cyp3a* expression in these species. Interestingly, the level of human ER proteins is partly regulated by activated AhR, which act as a ubiquitin ligase that selectively target sex steroid receptors for protein degradation^58^.

In order to further decipher the transcriptional regulation of Atlantic cod *cyp3a* and *cyp1a* orthologs in the absence of Pxr, we mapped the response elements in the 13 000 bp promoter region of these genes. The activated RXR/PXR heterodimer can bind several types of NR response elements, such as direct repeats (DR3, DR4, and DR5), inverted repeats (IR6), and everted repeats (ER6 and ER8)^59–62^. In humans, the active PXR response elements in the *CYP3A4* promoter are known to include a distal enhancer module containing a DR3 motif, a xenobiotic-responsive enhancer module (XREM) containing a DR3 and an ER6 motif, and a proximal ER6^63,64^. In addition, an HNF4α binding element (DR1/IR1) is important in transcriptional regulation of both human *CYP3A4* and mouse *Cyp3a11*^65,66^. Although the active PXR response elements are to some extent conserved between primates^34^, we did not find these in mouse *Cyp3a11* or fish *cyp3a* promoter regions. Notably, whereas the promotor regions of the mammalian *CYP3A4 and Cyp3a11* were dominated by NR response elements, the promoter regions of the teleost orthologs also included Ahr-binding xenobiotic response elements (XRE). In fact, we found that the relative number of XREs in the promoter region of cod *cyp3a* orthologs are higher than in zebrafish *cyp3a65*, where it is demonstrated that Ahr are necessary to mediate effective transcription of this gene^27^. Although the active XREs needed for transcription of human *CYP1A1*, mouse *Cyp1a1*, and zebrafish *cyp1a* are well described^35,37,67^, we were not able to recognize the position of these sites in the cod ortholog. However, similar to the promoter regions of cod *cyp3a166* and *cyp3a169*, we found that both the relative number of XREs, as well as the total number of response elements, in the cod *cyp1a* promoter region are higher than in the human, mouse, and zebrafish orthologs. Thus, transcriptional regulation of cod *cyp3a* genes appears more complex and may include extended coordinated signaling pathways and receptor crosstalk. This raises the question whether cod, and other fish lacking *pxr*, have a different response and sensitivity to environmental contaminants compared to fish that have retained the gene. When juvenile cod and rainbow trout (*Oncorhynchus mykiss*) were exposed in parallel to 2,3,7,8-tetrachlorodibenzo-*p*-dioxin (TCDD) – a potent agonist for the aryl hydrocarbon receptor, cod liver accumulated higher concentrations compared to trout, whereas that hepatic Cyp1a1 activity was lower^68^. Furthermore, exposing cod and turbot (*Scophthalamus maximus*) to two xenoestrogens, 4-nonylphenol and bisphenol A, showed indication of cod being the most sensitive and less specific in its response^69^. The genomes of trout and turbot were not included in our study, but whereas *pxr* is previously identified in rainbow trout^47^, we have not been able to clone this gene from turbot (unpublished results). Thus, it seems that there are inherent differences in how these fish species respond to chemicals that primarily interact with other receptor pathways, but, to our knowledge, a similar comparison aiming to activate the Pxr-pathway has not yet been published.

In addition to its xenosensing role, PXR has frequently been linked to functions in the immune system of mammals, such as co-regulation of CYPs^70–73^ and cross-talk with the inflammation and immune responding transcription factor NF-κB^74–76^. Interestingly, the initial sequencing of the Atlantic cod genome revealed the absence of several genes important for the adaptive immune system in other vertebrates, including the genes encoding the major histocompatibility complex (Mhc) II, Cd4 and invariant chain (II)^7,77^. Furthermore, low coverage genome sequencing of 66 teleost species demonstrated the absence of *mhcii* across the entire Gadiformes order^29^. However, whereas all Gadiformes, but no other fish genomes studied so far, lack *mhcii*^78^, our study show that the loss of *pxr* is not specific for the Gadiform order but has occurred several times during teleost evolution.

In conclusion, we present the first comprehensive evidence for evolutionary loss of the Pxr in teleost fish. Importantly, the evolutionary pattern strongly suggests that multiple independent losses of this important xenobiotic receptor have occurred early in the Gadiformes order, as well as in several other teleost lineages. Furthermore, we suggest that the Ahr has evolved a regulatory role for *cyp* gene transcription in Atlantic cod. The loss of *pxr*, and possible evolved compensatory mechanisms for the lack of this gene, adds to the scientific interest in Atlantic cod as a toxicological model species. However, as several independent losses have occurred in the course of teleost evolution, other lineages may have adapted other compensating mechanisms, which accentuate the need for understanding how the chemical defensome has evolved in fish.

## Material and Methods

### Searching 76 teleost species for a *pxr* gene

The selection of teleost genomes was performed based on the most robust and recent multi-genome phylogeny available at the time of the study^29,30^. This list includes 66 low-coverage genome assemblies^29,30^, including 27 species from the Gadiformes order and a newly published cod genome assembly^33^, and 10 other published teleost genomes (listed in Supplementary Table 13). Performing tBLASTn searches, the nucleotide sequence of the fish genome assemblies was searched using the amino acid sequence of Pxr from zebrafish (protein id. A5WYG8), medaka (*Oryzias latipes*, H2MUK7), tetraodon (*Tetraodon nigroviridis*, H3D8U6), and BXRβ from *Xenopus laevis* (Q9DF24) as query. To remove false positive BLAST hits that corresponded to the vitamin D receptor (Vdr, Nr1i1), we removed any BLAST hits that also was listed as Vdr from the same fish species (i.e. zebrafish Vdrα: Q9PTN2, medaka Vdr: H2MP78, and tetraodon Vdrα: H3CJ29) at a lower e-value. The output file of each search defined the coordinates (amino acid number for start and end) of the identified nucleotide segments (blast hits) in relation to the query Pxr sequence used. Based on these coordinates, it was counted how many times every position in the query Pxr sequence was overlapped by the identified segments, providing a coverage vector. The resulting coverage vectors of the BLAST hits identified from the Pxr of each sequence were assembled and visualized as a heat map, guided by the phylogenetic tree published in Malmstrøm *et al*.^29^. In these heat maps, one cell represents one amino acid in the Pxr sequence (x-axis) identified in the 76 different fish species (y-axis).

As a positive control of this approach, we applied this pipeline to search for the *mhcii* gene in the 76 fish genome assemblies, whose phylogenetic distribution in teleosts is recently characterized^29^.

Based on their ligand binding domains, sequences identified in the bioinformatic searches using zebrafish, medaka, and tetraodon Pxr as query were aligned with annotated or cloned teleost Pxr (Nr1i1), Vdr (Nr1i2), retinoic acid receptor (RARγ, Nr1b3) and Rev-ERbα (Nr1d1), using Clustal Omega v1.2.2 with default parameters (https://doi.org/10.6084/m9.figshare.6204920). Due to incomplete sequences in the LBD, *Cyttopsis roseus*, *Neoniphon samara*, *Lesueurigobius cf*., and *Holocentrus rupus* was excluded from the alignment. The maximum likelihood phylogeny was analyzed in RAxML v 8.1.14 using the CATLG model, with 200 bootstrap replicates, and drawn in FigTree v.1.4.2. The figure was prepared in Adobe Illustrator CS5.

### Syntenic relationship analysis

The genomic region containing the mammalian *PXR* gene is conserved, with *MAATS1* located immediately downstream and *GSK3B* immediately upstream of *PXR*. We used BLAST and Exonerate (EMBL-EMI) to search for these genes in 76 teleost genome assemblies^29^. Since the searches were performed in relatively fragmented genome assemblies, synteny was only revealed in the assemblies where the genes were located on the same scaffold. The phylogenetic relationships between the different teleost species were derived from Malmstrøm *et al*.^29^.

### Identifying nuclear receptors in the Atlantic cod genome

We identified the cod NR proteins in the Atlantic cod protein database (ENSEMBL-Gadus_morhua.gadMor.1.73, based on gadMor1 annotated proteins) by hidden Markov model (HMM) searches using HMMER v3.1b1 (www.hmmer.org) and the Pam profiles ‘Hormone_recep’ PF00104.25 and ‘zf-C4’ PF00105.13^79^. A Python script (available at https://doi.org/10.6084/m9.figshare.5752791) was used to perform the HMM searches, which were followed by reciprocal BLAST searches against the well-annotated zebrafish (*Danio rerio*) proteome (ENSEMBL-Danio_rerio.Zv9.73) to predict orthologous proteins. Based on the ENSEMBL protein identifiers, the corresponding gene identifiers were identified in the ENSEMBL databases and are presented in Supplementary Table 2.

Further, specific tblastn searches were also performed in the second version of the cod genome assembly (gadMor2; available at http://cees-genomes.hpc.uio.no/blast/33), using zebrafish Pxr (protein id. A5WYG8-1) as query sequence. The resulting gene entries were extracted and orthologs identified by reciprocal blastp searches in the UniProtKB database.

### Phylogenetic analyses of the NR-superfamily

A multiple sequence alignment of the Atlantic cod and zebrafish nuclear receptor proteins (available at https://doi.org/10.6084/m9.figshare.5752812) was constructed using ClustalX 2.1. Based on the resulting alignment, a phylogenetic tree was constructed using MrBayes v3.2.3 with the following settings: the prior for the amino acid model was set to mixed, and the number of generations used was 100 000^80^. FigTree v1.3.1 was used to visualize the tree, and the figure was prepared in Adobe Illustrator. For genes with predicted multiple protein sequences (splice variants), only the longest translation was included to ensure a non-redundant sequence set.

### Amplifying *pxr* from cod liver with consensus-degenerate hybrid oligonucleotide primers

Total RNA was extracted from liver tissue from two different cods using phenol and guanidinium isothiocyanate solution (Trizol, Thermo Fisher Scientific), before Superscript II Reverse transcriptase was used to synthesize cDNAs from total RNA as described by the supplier (Invitrogen). Consensus-degenerate hybrid oligonucleotide (CODEHOP) primers (Supplementary Table 3) were designed based on known Pxr sequences from eight fish species fish and human PXR (Supplementary Table 4) using the CODEHOP web tool^81^. The PCRs were performed using GoTaq DNA polymerase according to the protocol from the supplier (Promega) as gradient PCRs (T_anneal_: 46–60 °C).

### *Ex vivo* exposure to compounds known to activate mammalian and piscine PXR

Atlantic cod (*Gadus morhua*) was provided from Fjord Gadus AS (Fiskå, Norway), and maintained at The Industrial and Aquatic Research Laboratory (ILAB, Bergen, Norway). The fish were kept in 500 L tanks in natural seawater at 9 °C with a 12:12 hour light cycle regime. The fish were fed with a commercial diet *ad libitum* (EWOS, Bergen, Norway). Six immature cod of mixed sex, weighing 840–1549 g, was used in these experiments.

The fish were handled by FELASA C (Federation of European Laboratory Animal Science Association) approved personnel and killed according to the directions from the Norwegian Food Safety Authority (FOTS) § 16, second paragraph. Preparation of cod precision-cut liver slices (PCLS) was performed as described previously^10^. Freshly prepared cod PCLS were exposed for 24 hours to prototype PXR/Pxr-ligands described from other species, including clotrimazole, pregnenolone 16α-carbonitrile (PCN), rifampicin, and *p*-hydroxy benzoic acid (B4HB)^52^, as well as the well-known cod Ahr-ligand and *cyp1a*-inducer β-naphthoflavone (BNF). Chemicals were purchased from Sigma-Aldrich (Oslo, Norway) and dissolved in Hybri-Max™ dimethyl sulfoxide (DMSO, Sigma-Aldrich) to final concentrations of 0.25, 2.5, and 25 μM for PXR/Pxr-agonists, and 0.1, and 10 μM for BNF. 0.01% DMSO was used as solvent control in all exposures. Cytotoxicity of the chemicals was monitored by measuring the release of lactate dehydrogenase (LDH; EC 1.1.1.27) into the culture medium, using the LDH Cytotoxicity Detection Kit^PLUS^ (Roche Applied Sciences, Basel, Switzerland) according to the manufacturer’s protocol.

Total RNA was isolated from PCLS using the RNeasy Mini Kit (QIAGEN, Hilde, Germany). RNA concentration and RNA quality were assessed with a NanoDrop ND-1000 and agarose gel electrophoresis, and RNA was subsequently reverse transcribed to cDNA by either SuperScript III Reverse Transcriptase (Invitrogen, Thermo Fisher Scientific, Oslo, Norway) or qScript cDNA SuperMix (Quanta BioSciences, Gaithersburg, USA) following the producer’s protocol. The cDNA was diluted 1:30 in H_2_O prior to RT-qPCR using SYBR Green I Master (Roche) and the LightCycler® 480 Real-Time PCR System (Roche), according to the manufacturer’s procedure. Both a non-template control (H_2_O) and a positive control (calibrator cDNA) were included for each gene, and all samples were run in triplicates. The primers used for gene amplifications were synthesized by Sigma Aldrich (Supplementary Table 6). The transcriptional level of each gene was calculated using the Efficiency Calibrated Method of LightCycler® Software v1.5 (Roche), relating the expression to both a reference gene and a calibrator, and considering the efficiency of the reaction for each primer pair. Acidic ribosomal protein (*arp*) and ubiquitin (*ubi*) was used as reference genes^82^. Statistical analyses of RT-qPCR data were performed on Log_2_-converted relative gene expression in JMP v10 (SAS Institute) using Standard Least Squares with the fish individual as a random effect, followed by Tukey Kramer post hoc adjustment. Using this linear mixed-effects model, the variability of basal gene expression in individual fish is bypassed. Figures were prepared in Prism 6 (GraphPad), where the boxes represent the 25^th^ to 75^th^ percentile with the median indicated as a line and whiskers are drawn from the lowest to the highest value. Statistical significance (*p* < 0.05) between groups (control and two or three exposures groups) are marked by letters *a*, *b*, or *c*.

### Promoter analysis of *CYP3A* and *CYP1A* orthologs

Four orthologs to the zebrafish *cyp3a65* gene were identified in the ENSEMBL cod genome (‘gadMor1’) (Eide *et al*., *in prep*). These were confirmed in the new cod genome assembly^33^ and provided official names by the Cytochrome P450 Nomenclature Committee: *cyp3a166* (location in ‘gadMor2’: LG03, base pairs 18402807-18400860), *cyp3a167* (LG03, base pairs 18402807-18400860), *cyp3a168P* (LG03, base pairs 18384880-18386508), and *cyp3a169* (LG01, base pairs 14702701-14723855). Genomic regions containing 13,000 base pairs of upstream sequences of cod *cyp1a* (LG14, base pairs 2303196-2307638), *cyp3a166*, and *cyp3a169* were retrieved from the new genome assembly. The corresponding promoter regions of human *CYP3A4* (ENSG00000160868), *CYP1A1* (ENSG00000140465), and *CYP1A2* (ENSG00000140505); mouse *Cyp3a11* (ENSMUSG00000056035), *Cyp1a1* (ENMUSG00000032315), and *Cyp1a2* (ENSMUSG00000032310); and zebrafish *cyp3a65* (ENSDARG00000103295), and *cyp1a* (ENSDARG00000098315), were all derived from ENSEMBL.

Potential nuclear receptor response elements in the upstream regions were predicted using the nuclear hormone receptor (NHR)-scan available online^83^. NHR-scan uses a HMM framework for describing binding behavior according to three ‘match state chains’, corresponding to site configurations of direct repeats, inverted repeats, and everted repeats, compared to a background state^83^. We used the default settings with 0.01 probability of entering each of the match states. The extended Ahr-binding XREs were mapped by searching the sequences for the consensus sequence ‘KNGCGTGM’^37^ in forward, reverse and complementary directions. The resulting response elements were visualized in Adobe Illustrator CS5 (v15.0.0), and horizontal slice diagrams of binding site distributions were prepared in Prism 6 (GraphPad).

## Electronic supplementary material

Supplementary tables and figures
