# Supplementary material for: Independent losses of a xenobiotic receptor across teleost evolution
Source: Sci Rep. 2018 Jul 10;8:10404. doi: 10.1038/s41598-018-28498-4 (PMC6039460; doi:10.1038/s41598-018-28498-4)
Supplement: Supplementary file 1 — Supplementary tables and figures [file 41598_2018_28498_MOESM1_ESM.docx]

Supplementary information to

Independent losses of a xenobiotic receptor across teleost evolution

Marta Eide^1^, Halfdan Rydbeck^2^, Ole K. Tørresen^2^, Roger Lille-Langøy^1^, Pål Puntervoll^3^, Jared V. Goldstone^4^, Kjetill S. Jakobsen^2^, John Stegeman^4^, Anders Goksøyr^1^, and Odd André Karlsen*^1^

^1^ Department of Biological Sciences, University of Bergen, Bergen, Norway

^2^ Centre for Ecological and Evolutionary Synthesis (CEES), Department of Biosciences, University of Oslo, Oslo, Norway

^3^ Centre for Applied Biotechnology, Uni Research Environment, Bergen, Norway

^4^ Biology Department MS, Woods Hole Oceanographic Institution, Woods Hole, MA, USA

* Corresponding author: odd.karlsen@uib.no

**Additional files**

The following supplementary figure and tables are available in this document:

- Figure I shows the results from BLAST searches in 76 teleost genomes using medaka and tetraodon Pxr as query.
- Figure II shows the results from BLAST searches in 76 teleost genomes using zebrafish Vdrα as query.
- Figure III shows the positional mapping of response elements in promoter regions of CYP3A and CYP1A orthologs
- Table I lists the teleost genome assemblies that was used to search for the *pxr* gene
- Table II is an overview of the nuclear receptors identified in Atlantic cod and zebrafish
- Table III shows the CODEHOP *pxr* primers used for PCR, of which the sequences was based on the species listed in Table IV. The blastn hits from the resulting cloned sequences are shown in Table V
- Table VI shows the primers used for quantitative PCR
- Table VII specifies the number of NR response elements and XREs identified in the promoter regions of *CYP3A* and *CYP1A* orthologs.

In addition, the following files are available for download from the online sites:

- Multiple sequence alignment of teleost Pxr sequences: https://doi.org/10.6084/m9.figshare.6204920.
- Python script: https://doi.org/10.6084/m9.figshare.5752791.
- Multiple sequence alignment of Atlantic cod and zebrafish NR sequences: https://doi.org/10.6084/m9.figshare.5752812.

**Supplementary figures**


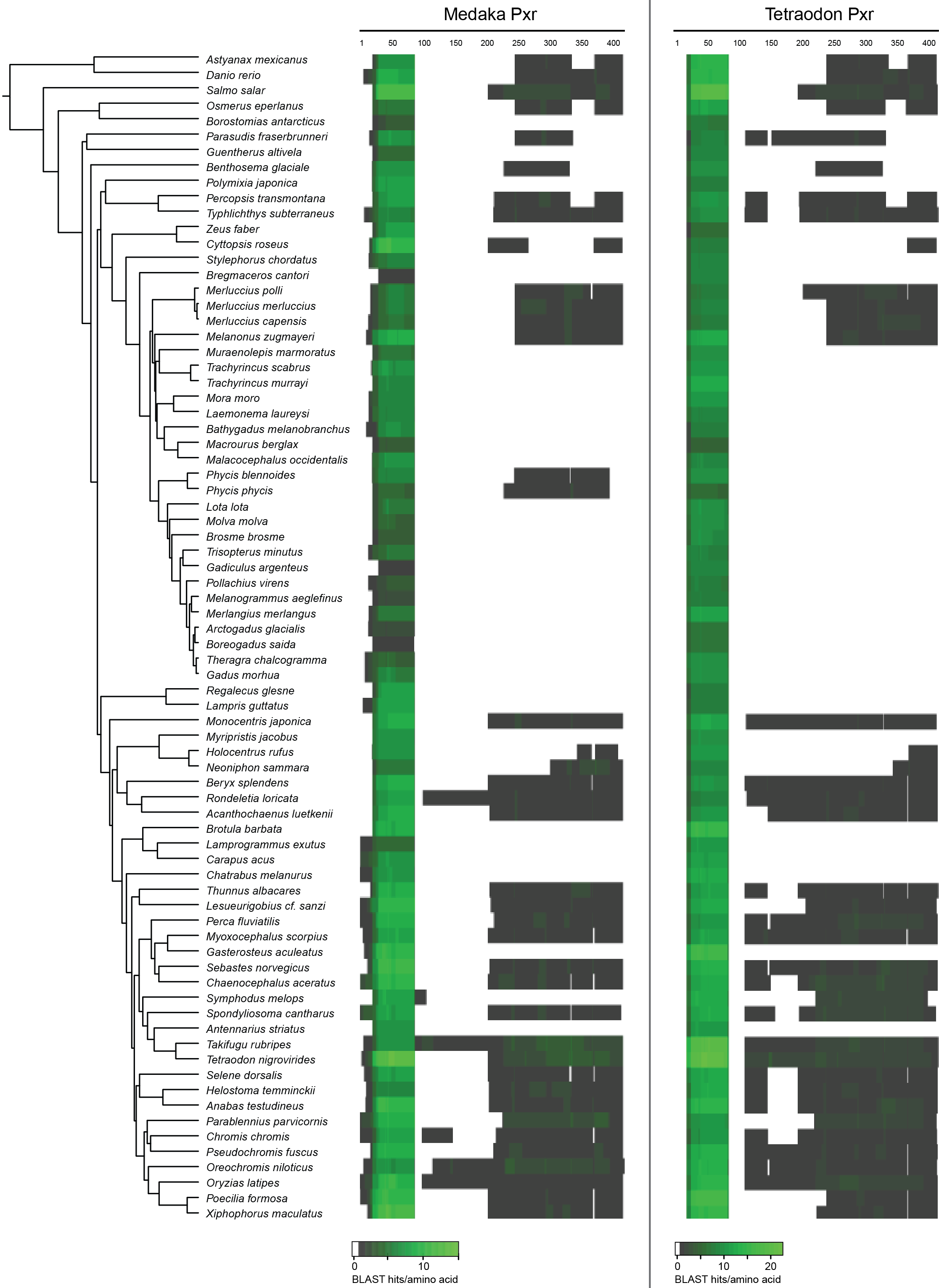


**Figure I: *In silico* searches using pregnane x receptor (Pxr) from medaka (*Oryzias latipes*, 414 amino acids) and tetraodon (*Tetraodon nigroviridis*, 404 amino acids).** Each row show assembly of the resulting coverage vectors of the BLAST hits identified as *pxr* in 76 different fish species. For each amino acid, the colors indicate one single BLAST hit (dark green), or several BLAST hits (light green). The map is guided by the phylogenetic tree published in Malmstrøm, M., et al. (2016). "Evolution of the immune system influences speciation rates in teleost fishes." Nature Genetics **48**(10): 1204-1210.

.


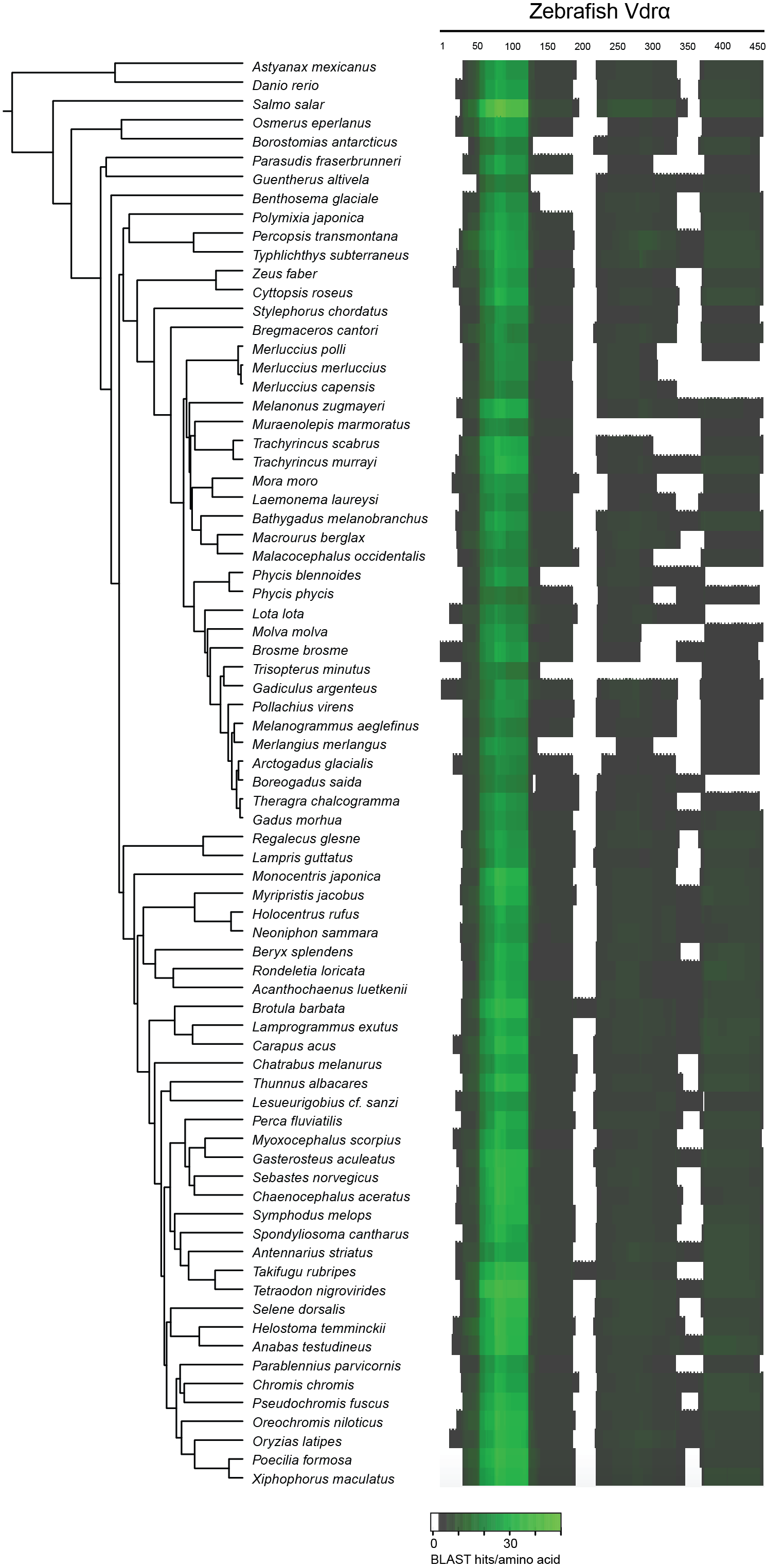


**Figure II: *In silico* searches using vitamin D receptor (Vdrα, Nr1i1) from zebrafish (*Danio rerio*, 453 amino acids).** Each row show assembly of the resulting coverage vectors of the BLAST hits identified as *pxr* in 76 different fish species. For each amino acid, the colors indicate one single BLAST hit (dark green), or several BLAST hits (light green). The map is guided by the phylogenetic tree published in Malmstrøm, M., et al. (2016). "Evolution of the immune system influences speciation rates in teleost fishes." Nature Genetics **48**(10): 1204-1210.


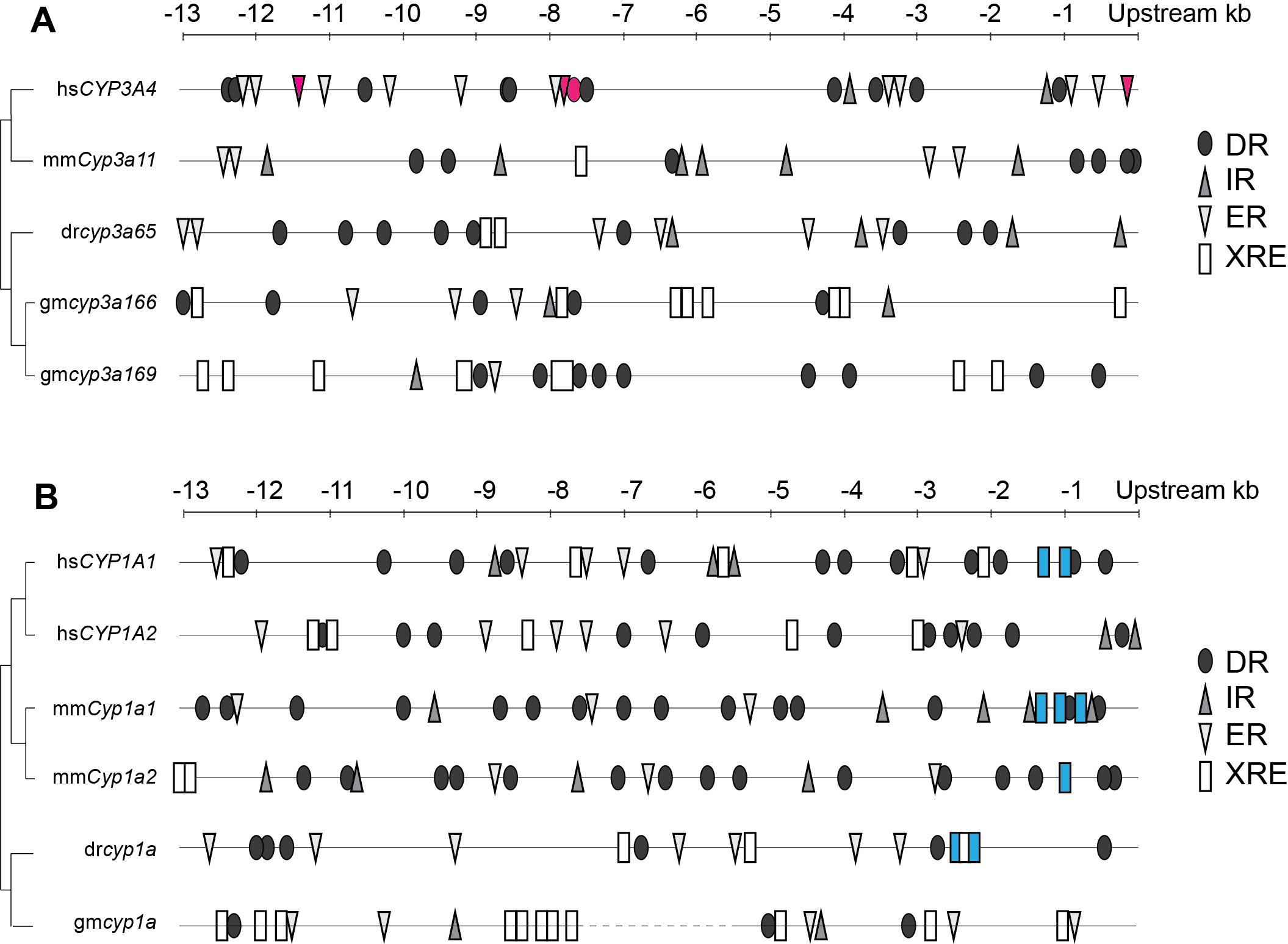


**Figure III: Positional mapping of response elements (REs) in CYP3A orthologs (A), CYP1A orthologs (B) in 13 kb upstream regions of CYP3A and CYP1A orthologs.** Species included are humans (hs), mouse (mm), zebrafish (*Danio rerio*, dr), and Atlantic cod (*Gadus morhua*, gm). Potential nuclear receptor response elements were revealed using NHR-Scan, and the AHR-binding xenobiotic response elements (XREs) were identified by searching for the consensus sequence ‘KNGCGTGM’. Based on current literature, known PXR binding sites are indicated in pink (A), and known XREs in blue (B). A gap of ~2,000 base pairs where the sequence is not available is indicated in gm*cyp1a* by a dotted line.

**Supplementary tables**

**Table I: List of teleost genomes used to search for the *pxr* gene.** 66 of the fish genome assemblies were recently published in Malmstrøm et al. 2016, whereas the last 9 teleost genome assemblies are publicly available in ENSEMBL (indicated by asterisk).

| **Order** | **Family** | **Species** | **Common name** |
| --- | --- | --- | --- |
| Ateleopodiformes | Ateleopodidae | *Guentherus altivela* | Jellynose |
| Aulopiformes | Chlorophthalmidae | *Parasudis fraserbrunneri* | Tripodfish |
| Aulopiformes | Synodontidae | *Saurida brasiliensis* | Brazilian lizardfish |
| Aulopiformes | Synodontidae | *Synodus synodus* | Diamond lizardfish |
| Batrachoidiformes | Batrachoididae | *Chatrabus melanurus* | NA |
| Batrachoidiformes | Batrachoididae | *Perulibatrachus rossignoli* | Rossignol toadfish |
| Batrachoidiformes | Batrachoididae | *Opsanus beta* | Gulf toadfish |
| Beloniformes | Adrianichthyidae | *Oryzias latipes** | Medaka |
| Beryciformes | Holocentridae (S) | *Neoniphon sammara* | NA |
| Beryciformes | Monocentridae | *Monocentris japonica* | NA |
| Beryciformes | Holocentridae | *Myripristis jacobus* | Blackbar soldierfish |
| Beryciformes | Holocentridae | *Holocentrus rufus* | Longspine squirrelfish |
| Beryciformes | Trachichthyidae | *Gephyroberyx darwini* | Darwinian slimehead |
| Beryciformes | Berycidae | *Beryx splendens* | NA |
| Beryciformes | Anoplogastridae | *Anoplogaster cornuta* | NA |
| Beryciformes | Diretmidae | *Diretmus argenteus* | NA |
| Beryciformes | NA | *Diretmoides pauciradiatus* | NA |
| Beryciformes | Trachichthyidae (S) | *Gephyroberyx darwiniÊ* | NA |
| Beryciformes | NA | *Hoplostethus atlanticus* | NA |
| Cetomimiformes | Cetomimidae | *Cetomimus sp* | NA |
| Cetomimiformes | Rondeletiidae | *Rondeletia loricata* | NA |
| Characiformes | Characidae | *Astyanax mexicanus** | NA |
| Cypriniformes | Cyprinidae | *Danio rerio** | Zebrafish |
| Cyprinodontiformes | Poeciliidae | *Poecilia formosa** | Amazon molly |
| Cyprinodontiformes | Poeciliidae | *Xiphophorus maculatus** | NA |
| Gadiformes | Gadidae | *Arctogadus glacialis* | Arctic cod |
| Gadiformes | Gadidae | *Boreogadus saida* | Polar cod |
| Gadiformes | Gadidae | *Trisopterus minutus* | Poor cod |
| Gadiformes | Gadidae | *Pollachius virens* | Saithe |
| Gadiformes | Gadidae | *Melanogrammus aeglefinus* | Haddoc |
| Gadiformes | Gadidae | *Merlangius merlangus* | Whiting |
| Gadiformes | Gadidae | *Theragra chalcogramma* | Alaska pollock |
| Gadiformes | Gadidae | *Gadiculus argenteus* | Silvercod |
| Gadiformes | Phycidae | *Phycis phycis* | Forkbeard |
| Gadiformes | Lotidae | *Molva molva* | Ling |
| Gadiformes | Lotidae | *Lota lota* | Burbot |
| Gadiformes | Lotidae | *Brosme brosme* | Cusk |
| Gadiformes | Merlucciidae | *Merluccius merluccius* | Hake |
| Gadiformes | Merlucciidae | *Merluccius capensis* | Shallow-water Cape hake |
| Gadiformes | Merlucciidae | *Merluccius polli* | Black hake |
| Gadiformes | Melanonidae | *Melanonus zugmayeri* | Arrowtail |
| Gadiformes | Macrouridae | *Macrourus berglax* | Roughhead grenadier |
| Gadiformes | Macrouridae | *Malacocephalus occidentalis* | Western softhead grenadier |
| Gadiformes | Bathygadidae | *Bathygadus melanobranchus* | Vaillants grenadier |
| Gadiformes | Muraenolepididae | *Muraenolepis marmoratus* | Marbled moray cod |
| Gadiformes | Moridae | *Mora moro* | Common mora |
| Gadiformes | Moridae | *Laemonema laureysi* | Guinean codling |
| Gadiformes | Macrouridae | *Trachyrincus scabrus* | Roughsnout grenadier |
| Gadiformes | Raniceptidae | *Raniceps raninus* | Tadpolefish |
| Gadiformes | Macrouridae | *Coryphaenoides rupestris* | Roundnose grenadier |
| Gadiformes | Macrouridae | *Trachyrincus murrayi* | Murray’s longsnout grenadier |
| Gadiformes | Phycinae | *Phycis blennoides* | Greater forkbeard |
| Gadiformes | Gadidae | *Gadus morhua* | NEAC_001 |
| Gadiformes | Bregmacerotidae | *Bregmaceros cantori* | Striped codlet |
| Gasterosteiformes | Gasterosteidae | *Gasterosteus aculeatus* | Three-spined stickleback |
| Lampriformes | Trachypteridae | *Regalecus glesne* | King of herring |
| Lampriformes | Trachypteridae | *Lampris guttauts* | Opah |
| Lampriformes | Lophotidae | *Lophotus capellei* | Unicornfish |
| Lophiiformes | Lophiidae | *Lophius vaillanti* | Shortspine African angler |
| Lophiiformes | Antennariidae | *Antennarius striatus* | Striated frogfish |
| Myctophiformes | Myctophidae | *Benthosema glaciale* | NA |
| Notothenioidei | Eleginopsidae | *Eleginops maclovinus* | Patagonian blennie |
| Notothenioidei | Nototheniidae | *Patagonotothen guntheri* | Yellowfin notothen |
| Notothenioidei | Nototheniidae | *Trematomus newnesi* | Dusky rockcod |
| Notothenioidei | Nototheniidae | *Pleuragramma antarctica* | Antarctic silverfish |
| Notothenioidei | Channichthyidae | *Chaenocephalus aceratus* | Blackfin icefish |
| Notothenioidei | Artedidraconidae | *Artedidraco skottsbergi* | Antarctic plunderfish |
| Notothenioidei | Harpagiferidae | *Harpagifer kerguelensis* | Kerguelen spiny plunderfish |
| Notothenioidei | Bathydraconidae | *Gymnodraco acuticeps* | Ploughfish |
| Ophidiiformes | Ophidiidae | *Brotula barbata* | Bearded brotula |
| Ophidiiformes | Ophidiidae | *Lamprogrammus exutus* | Legless cusk eel |
| Ophidiiformes | Carapidae | *Carapus acus* | Pearl fish |
| Osmeriformes | Osmeridae | *Osmerus eperlanus* | European smelt |
| Perciformes | Cichlidae | *Oreochromis niloticus** | Nile tilapia |
| Perciformes | Percidae | *Perca fluviatilis* | European perch |
| Perciformes | Scombridae | *Thunnus albacares* | Yellowfin tuna |
| Perciformes | Anabantidae | *Anabas testudineus* | NA |
| Perciformes | Carangidae | *Selene dorsalis* | NA |
| Perciformes | Gobiidae | *Lesueurigobius cf. sanzoi* | NA |
| Perciformes | Sparidae | *Spondyliosoma cantharus* | NA |
| Perciformes | Gobiidae | *Rhyacichthys aspro* | NA |
| Perciformes | Helostomatidae | *Helostoma temminckii* | NA |
| Perciformes | Pomacentridae | *Chromis chromis* | NA |
| Perciformes | Blenniidae | *Parablennius parvicornis* | NA |
| Perciformes | Labridae | *Symphodus melops* | NA |
| Perciformes | Pseudochromidae | *Pseudochromis fuscus* | NA |
| Percopsiformes | Percopsidae | *Percopsis transmontana* | Sandroller |
| Percopsiformes | Amblyopsidae | *Typhlichthys subterraneus* | Southern cavefish |
| Polymyxiformes | Polymixidae | *Polymixia japonica* | Silver eye beardfish |
| Salmoniformes | Salmonidae | *Salmo salar** | Atlantic salmon |
| Scorpaeniformes | Sebastidae | *Sebastes norvegicus* | Golden redfish |
| Scorpaeniformes | Cottidae | *Myoxocephalus scorpius* | NA |
| Stephanoberyciformes | Melamphaidae | *Melamphaidae sp.* | NA |
| Stephanoberyciformes | Melamphaidae (S) | *Poromitra crassiceps* | NA |
| Stephanoberyciformes | NA | *Scopelogadus beanii* | NA |
| Stephanoberyciformes | Stephanoberycidae | *Acanthochaenus luetkenii* | NA |
| Stomiformes | Stomiidae | *Borostomias antarcticus* | NA |
| Stylephoriformes | Stylephoridae | *Stylephorus chordatus* | Tube-eye or thread-tail |
| Tetraodontiformes | Tetraodontidae | *Takifugu rubripes** | NA |
| Tetraodontiformes | Tetraodontidae | *Tetraodon nigroviridis** | NA |
| Zeiformes | Zeidae | *Zeus faber* | John dory |
| Zeiformes | Zeidae | *Cyttopsis roseus* | Red dory |
| Zeiformes | Zeidae | *Zenopsis conchifer* | Silvery john dory |

Table II: Overview of nuclear receptors identified in Atlantic cod and zebrafish genomes by HMM-searches using relevant Pfam-profiles. Protein IDs of the longest transcripts are shown for cod and zebrafish, and the corresponding cod gene. Grey letters mark the nuclear receptors not considered relevant for the chemical defensome.

|  | | **ENSEMBL proteins used in phylogenetic tree** | |
| --- | --- | --- | --- |
| **Gene** | **NR sub-family** | **Atlantic cod (_gm)** | **Zebrafish (_dr)** |
|  |  |  |  |
| *thraa* | *nr1a1* | ENSGMOP00000002248 | ENSDARP00000000160 |
| *thrab* |  | ENSGMOP00000017305 | ENSDARP00000069037 |
| *thrb* | *nr1a2* | ENSGMOP00000008617 | ENSDARP00000126275 |
| *raraa* | *nr1b1* | ENSGMOP00000010526 | ENSDARP00000073792 |
| *rarab* |  | ENSGMOP00000002797 | ENSDARP00000111644 |
| *rarb* | *nr1b2* | ENSGMOP00000020616 |  |
|  |  | ENSGMOP00000010516 |  |
| *rarga* | *nr1b3* | ENSGMOP00000019164 | ENSDARP00000093932 |
|  |  | ENSGMOP00000002445 |  |
| *rargb* |  |  | ENSDARP00000052545 |
| *pparaa* | *nr1c1* | ENSGMOP00000006307 | ENSDARP00000042466 |
| *pparab* |  | ENSGMOP00000001101 | ENSDARP00000070972 |
|  |  |  | ENSDARP00000105666 |
| *ppapda* | *nr1c2* |  | ENSDARP00000109331 |
| *ppardb* |  | ENSGMOP00000008795 | ENSDARP00000110137 |
| *pparg* | *nr1c3* | ENSGMOP00000001443 | ENSDARP00000039949 |
| *rev-erba* | *nr1d1* | ENSGMOP00000012307 | ENSDARP00000104933 |
|  | *nr1d2a* | ENSGMOP00000020628 | ENSDARP00000089813 |
|  | *nr1d2b* | ENSGMOP00000008612 | ENSDARP00000112360 |
|  | *nr1d4a* | ENSGMOP00000005632 | ENSDARP00000093945 |
|  | *nr1d4b* | ENSGMOP00000019693 | ENSDARP00000076910 |
| *roraa* | *nr1f1* | ENSGMOP00000007376 | ENSDARP00000106236 |
| *rorab* |  | ENSGMOP00000000830 | ENSDARP00000090251 |
| *rorb* | *nr1f2* | ENSGMOP00000017693 | ENSDARP00000084181 |
| *rorc* | *nr1f3* | ENSGMOP00000008579 | ENSDARP00000124002 |
| *rorca* |  | ENSGMOP00000000786 | ENSDARP00000091257 |
| *rorcb* |  | ENSGMOP00000013141 | ENSDARP00000105771 |
| *lxr* | *nr1h3* | ENSGMOP00000005729 | ENSDARP00000108778 |
| *fxr* | *nr1h4* | ENSGMOP00000004002 | ENSDARP00000061793 |
|  | *nr1h5* | ENSGMOP00000017126 | ENSDARP00000064039 |
| *vdr* | *nr1i1* | ENSGMOP00000020924* | ENSDARP00000063213 |
| *vdrb* |  | ENSGMOP00000013607* | ENSDARP00000125655 |
| *pxr* | *nr1i2* |  | ENSDARP00000106185 |
| *hnf4a* | *nr2a1* | ENSGMOP00000012211 | ENSDARP00000112924 |
|  |  | ENSGMOP00000003287 |  |
| *hnf4g* | *nr2a2* | ENSGMOP00000002105 | ENSDARP00000120982 |
| *rxraa* | *nr2b1* | ENSGMOP00000015513 | ENSDARP00000104454 |
| *rxrab* |  |  | ENSDARP00000004918 |
| *rxrba* | *nr2b2* | ENSGMOP00000011152 | ENSDARP00000119017 |
| *rxrbb* |  | ENSGMOP00000010373 | ENSDARP00000104696 |
| *rxrga* | *nr2b3* | ENSGMOP00000019379 | ENSDARP00000021935 |
| *rxrgb* |  |  | ENSDARP00000003080 |
| *tr2* | *nr2c1* | ENSGMOP00000019690 | ENSDARP00000066947 |
| *tr4* | *nr2c2* | ENSGMOP00000018317 | ENSDARP00000107308 |
| *tlx* | *nr2e1* | ENSGMOP00000017194 | ENSDARP00000013317 |
| *pnr* | *nr2e3* | ENSGMOP00000020640 | ENSDARP00000111061 |
|  |  | ENSGMOP00000008924 |  |
| *coup-tfia* | *nr2f1* | ENSGMOP00000000785 | ENSDARP00000116750 |
| *coup-tfib* |  |  | ENSDARP00000010118 |
| *coup-tfii* | *nr2f2* | ENSGMOP00000016617 | ENSDARP00000059975 |
|  |  | ENSGMOP00000015548 |  |
|  | *nr2f5* | ENSGMOP00000016025 | ENSDARP00000040768 |
| *ear-2a* | *nr2f6* | ENSGMOP00000011070 | ENSDARP00000009513 |
| *ear-2b* |  |  | ENSDARP00000111987 |
| *esr1* | *nr3a1* | ENSGMOP00000015968 | ENSDARP00000024987 |
| *esr2a* | *nr3a2* | ENSGMOP00000020435 | ENSDARP00000110930 |
| *esr2b* |  | ENSGMOP00000000804 | ENSDARP00000041299 |
| *esrra* | *nr3b1* | ENSGMOP00000015776 | ENSDARP00000091431 |
| *esrrb* | *nr3b2* | ENSGMOP00000016257 | ENSDARP00000008527 |
| *esrrd* |  | ENSGMOP00000011891 | ENSDARP00000106954 |
| *esrrga* | *nr3b3* | ENSGMOP00000003584 | ENSDARP00000111028 |
| *esrrgb* |  | ENSGMOP00000001656 | ENSDARP00000091109 |
| *gr* | *nr3c1* | ENSGMOP00000006025 | ENSDARP00000054263 |
|  |  | ENSGMOP00000019143 |  |
| *mr* | *nr3c2* | ENSGMOP00000002015 | ENSDARP00000104593 |
| *pgr* | *nr3c3* | ENSGMOP00000003076 | ENSDARP00000109370 |
| *ar* | *nr3c4* | ENSGMOP00000010954 | ENSDARP00000088795 |
|  |  | ENSGMOP00000004335 |  |
| *ngfib* | *nr4a1* | ENSGMOP00000016296 | ENSDARP00000001911 |
|  |  | ENSGMOP00000007805 |  |
| *nurr1a* | *nr4a2* | ENSGMOP00000020670 | ENSDARP00000100256 |
| *nurr1b* |  | ENSGMOP00000004008 | ENSDARP00000065384 |
| *nor1* | *nr4a3* | ENSGMOP00000015497 | ENSDARP00000072712 |
| *sf1* | *nr5a1* | ENSGMOP00000010010 | ENSDARP00000114497 |
|  |  | ENSGMOP00000007253 | ENSDARP00000029204 |
| *lrh-1* | *nr5a2* | ENSGMOP00000001189 | ENSDARP00000106755 |
| *ff1c* | *nr5a5* | ENSGMOP00000009827 | ENSDARP00000105488 |
| *gcnfa* | *nr6a1* | ENSGMOP00000009407 | ENSDARP00000105986 |
| *gcnfb* |  |  | ENSDARP00000109637 |
| *dax-1* | *nr0b1* | ENSGMOP00000018057 | ENSDARP00000105523 |
| *shp* | *nr0b2* | ENSGMOP00000001008 | ENSDARP00000065659 |
|  |  |  | ENSDARP00000008536 |

*For the two Vdr paralogs in Atlantic cod, we supplemented the alignment with cloned sequences from Ueland *et al*., (personal communication).

**Table III: Primers used for consensus-degenerate hybrid oligonucleotide primers PCR**. Primers were designed to recognize conserved regions of teleost pregnane x receptor (*pxr)* genes and used in various combinations in attempts to amplify gene sequences.

| **Primer name** | **Sequence (5’-3’)** |
| --- | --- |
| PXR_fish_dbd_fwdA | CTACCACTTCAACGCCATGACNTGYGA |
| PXR_fish_lbd_revH | CAGCACCTGCTTGGTGTACTCYTCRTTCAT |
| PXR_fish_lbd_wdB | AAGAGGGAGATGATCATGTCCRANGARGMNGT |
| PXR_fish_lbd_revE | GCCCTTCAGCAGGGAGATYTGRTCNTC |
| PXR_fish_lbd_fwdE | TCACCGACCTGACCACCTAYATGATHM |
| PXR_fish_lbd_revC | TCCTGGATCACCGCCTCYTGYTGNGG |

**Table IV: Overview of PXR/Pxr sequenced used in design of consensus-degenerate hybrid oligonucleotide primers used in attempts to amplify teleost *pxr* gene orthologs.**

| **Common name** | **Systematic name** | **Accession number** |
| --- | --- | --- |
| Zebrafish | *Danio rerio* | NP_001092087 |
| Medaka/Japanese rice fish | *Oryzias latipes* | ABV29345 |
| Fathead minnow | *Pimephales promelas* | ABV29431 |
| Atlantic salmon | *Salmo salar* | Personal communications: Rune Male, University of Bergen, Norway |
| Rainbow trout | *Oncorhynchus mykiss* | NP_001118144 |
| Mummichog/Killifish | *Fundulus heteroclitus* | ABR21208 |
| Green spotted pufferfish | *Tetraodon_nigroviridis* | CAG05861 |
| Japanese pufferfish/fugu | *Takifugu rubripes* | ABV29342 |
| Human | *Homo sapiens* | O75469 |

**Table V: Results from polymerase chain reaction (PCR) using** **consensus-degenerate hybrid oligonucleotide primer #977 in fish liver tissues**

| **Common name** | **Systemic name** | **Sequenced product length (bp)** | **Top blastn hit** | **Accession** | **E value** |
| --- | --- | --- | --- | --- | --- |
| European hake | *Merluccius merluccius* | 840 | PREDICTED: Salmo salar nuclear receptor subfamily 1 group I member 2-like (LOC106586651), transcript variant X3, mRNA | XM_014174164.1 | 4e-18 |
| Atlantic herring | *Clupea harengus* | 903 | PREDICTED: Clupea harengus nuclear receptor subfamily 1, group I, member 2 (nr1i2), mRNA | XM_012831884.1 | 0.0 |
| European eel | *Anguilla anguilla* | 1042 | PREDICTED: Calidris pugnax nuclear receptor subfamily 1 group I member 3 (NR1I3), mRNA | XM_014961882.1 | 1e-24 |
| Atlantic wolffish | *Anarhichas lupus* | 994 | PREDICTED: Larimichthys crocea nuclear receptor subfamily 1 group I member 2 (nr1i2), transcript variant X1, mRNA | XM_010752971.2 | 0.0 |

**Table VI: Primers used for Real-time quantitative PCR (RT-qPCR) in Atlantic cod**. Gene short names, GenBank accession number, primer sequences and amplicon sizes are shown.

| **Gene name (ENSEMBL)** | **Accession no.** | **Forward primer (5’-3’)** | **Reverse primer (5’-3’)** | **Product size (bp)** |
| --- | --- | --- | --- | --- |
| *arp^a^* | EX741373 | TGATCCTCCACGACGATGAG | CAGGGCCTTGGCGAAGA | 113 |
| *cyp1a^b^* | ENSGMOG00000000318 | CACCAGGAGATCAAGGACAAG | GCAGGAAGGAGGAGTGACGGAA | 118 |
| *cyp3a166* | ENSGMOG00000007792 | GCATACAAACGAGGCTTTACC | TGAGGGAGACATCTGTGGTG | 115 |
| *cyp3a169* | ENSGMOG00000003387 | CAACAGACGAGACTTTGGCTTG | CGCTGGTAAACGATGGAGAC | 110 |
| *ubi^a^* | EX735613 | GGCCGCAAAGATGCAGAT | CTGGGCTCGACCTCAAGAGT | 69 |

^a^ Olsvik, P. A., et al. (2008). "Selection of reference genes for qRT-PCR examination of wild populations of Atlantic cod Gadus morhua." BMC Research Notes **1**: 47.

^b^ Yadetie F. (personal communication)

**Table VII: Overview of the number of XREs and NR response elements (direct, inverted and everted repeats) in the promoter region of mammalian and teleost orthologs of CYP3A and CYP1A**

|  | **Direct repeats** | **Inverted repeats** | **Evereted repeats** | **Total NR REs** | **XREs**  **(KNGCGTGM)** | **Total number of REs** |
| --- | --- | --- | --- | --- | --- | --- |
|  |  |  |  |  |  |  |
| hm_*CYP3A4* | 12 | 2 | 13 | 27 | 0 | 27 |
| mm_*CYP3A11* | 7 | 6 | 4 | 17 | 1 | 18 |
| dr_*cyp3a65* | 9 | 4 | 6 | 19 | 8 | 27 |
| gm_*cyp3a166* | 6 | 2 | 3 | 11 | 13 | 24 |
| gm_*cyp3a169* | 11 | 1 | 1 | 13 | 65 | 78 |
|  |  |  |  |  |  |  |
| hs_*CYP1A1* | 14 | 3 | 5 | 22 | 12 | 34 |
| hm_*CYP1A2* | 12 | 2 | 8 | 22 | 5 | 27 |
| mm_*Cyp1a1* | 19 | 6 | 3 | 28 | 6 | 34 |
| mm_*Cyp1a2* | 19 | 5 | 3 | 27 | 5 | 32 |
| dr_*cyp1a* | 7 | 0 | 7 | 14 | 8 | 22 |
| gm_*cyp1a* | 3 | 2 | 5 | 10 | 18 | 28 |
